# Supplementary material for: A Unique Case of Bilateral Thalamic High-Grade Glioma in a Pediatric Patient with LI-Fraumeni Syndrome: Case Presentation and Review of the Literature
Source: Neurol Int. 2021 Apr 22;13(2):175–83. doi: 10.3390/neurolint13020017 (PMC8167566; doi:10.3390/neurolint13020017)
Supplement: Supplementary file 1 [file neurolint-13-00017-s001.zip › neurolint-1095973-supplementary.pdf]

No	Item	Guide and description
1	Aim	A unique case of bilateral Thalamic High-Grade Glioma in a pediatric patient with Li-Fraumeni Syndrome: Case Presentation and Review of the Literature *(Title)*
2	Synthesis methodology	Case report and qualitative systematic review of high-grade gliomas in LFS in pediatric population reported in the literature
3	Approach to searching	This was a pre-planned comprehensive search of all cases published in the literature
4	Inclusion criteria	Inclusion criteria<br>- Population: pediatric population only (from newborn to 18 years old)<br>- Language: English<br>- Year limits: from January 2000 to November 2020<br>- Type of publication: peer reviewed articles, full text-paper<br>- Study type: case report, case series, review, research study<br>Exclusion criteria<br>- Population: Adult population and young adults (above 18 years old)<br>- Year limits: before December 1999<br>- Type of publication: abstract, non peer reviewed articles
5	Data sources	Electronic database MEDLINE/Pubmed and MeSH
6	Electronic Search strategy	The literature search included two clinical terms: "Li-Fraumeni Syndrome" and "pediatric glioma"<br>Search limits were clinical series including both pediatric and adult patients, low-grade and high-grade glioma
7	Study screening methods	R.M., T.P. and V.S. reviewed the literature, screened the abstracts of the reference list, deleted duplicates and citations not meeting the inclusion criteria, and assessed the articles; G.C. and V.B. wrote the case presentation; G.I. and G.C. performed the pathologic findings and the genetic analysis; M.R. elaborated the images and prepared the captions; R.M. and T.P. wrote the manuscript; R.M and V.S. prepared the review in line with the ENTREQ Statement using a 21-point checklist. The first version of the article was submitted to F.D.L and N.S., who contributed to perform the discussion; F.S. critically revised the manuscript for intellectual content. Through an iterative approach and an analysis of the pertinent literature, the corrected version of the article was discussed collegially and a final version was produced *(Author's contributions).*
8	Study characteristics	Included studies:<br>1. Case report, 2020, Ecuador, genomic characterization of rare disease<br>2. Population-based study, 2020, United States, germline cancer predisposition variants review<br>3. Clinical series, 2020, United States, 13 patients with glioma arising in the context of Li-Fraumeni Syndrome<br>4. Case report, 2019, United States, precision medicine from multidisciplinary team targeting rare cases<br>Year of publication:<br>• All the articles included in this review were published between 2019 and 2020,<br>Country, population:<br>• USA and Ecuador<br>Data collection, methodology, analysis:<br>• 2 Case report, 1 Clinical series, 1 Population-based study<br>Research questions:<br>• rare cancer disease report in the context of Li-Fraumeni Syndrome
9	Study selection results	Studies identified via database searching: 32<br>Sudies identified via Pubmed: 21<br>Studies identified via MeSH: 11<br>Study exclusion: 11 duplicates; 18 not relevant to the research subtype<br>*(Figure S1. Flowchart of search strategy used in the study)*
10	Rationale for appraisal	All pediatric cases of high-grade glioma arising in the context of Li-Fraumeni
11	Appraisal items	Mixed methods approach: comprehensive syntheses of quantitative and qualitative data issue from case series, reviews and research studies published between January 2000 and November 2020 in peer-reviewed journals was conducted and combined into a final synthesis
12	Appraisal process	The appraisal was conducted independently by more than one reviewer and consensus gained by other Authors.

13	Appraisal results	We identified a total of 32 records through database searching according to our selection criteria (full-text only articles published from January 2000 to November 2020). Twenty-one publications were obtained through MEDLINE/PubMed and 11 from MeSH Database. Eleven studies were duplicate and were excluded from our review. A total of 21 peer-reviewed papers were screened based on this review's focus (HGG in pediatric patients with LFS). Eighteen papers were found not relevant and were excluded, leaving only 3 studies to be included in our qualitative analysis (Figure S1). A total of 6 cases of pediatric patients with LFS and brain HGGs were identified *(Table 1)*.
14	Data extraction	All the patients discussed in the result section were individually selected from the 4 articles (point 8) and grouped in Tab1. Our patient was also added to Tab1. Only high-grade gliomas in pediatric patient with LFS were selected.
15	Software	No statistical analysis was required for this research
16	Number of reviewers	2
17	Coding	Authors evaluated all sections of included articles and filled a coded data extraction form that included reference of the included article, number of cases included and, for each case, age at diagnosis, cancer history, tumor histology and immunohistochemistry, tumor location, signs and symptoms, treatment, outcome
18	Study comparison	Genetic mutations, Treatment modality and Survival rate were compared in the patient cohort obtained across studies
19	Derivation of themes	Themes were identified through an inductive process from Abstract analysis and, when searching from missing information, from full-text
20	Quotations	"*Next-generation sequencing allows genomic characterization of rare diseases to be implemented in routine clinical practice for early detection and effective preemptive intervention delivery and treatment*"<br><br>"*A considerable fraction of pediatric glioma patients, especially those of higher grade, harbor a putatively pathogenic variant in a cancer predisposition gene. Some of these variants may be clinically actionable or may warrant genetic counseling*"<br><br>"*Genomic characterization study and clinic-pathologic features of gliomas arising in the setting of LFS*"
21	Synthesis output	*(Conclusions)*
